# Unearthing a legacy from the green revolution: *Rht-D1b* contributes to larger roots in modern bread wheat varieties

**DOI:** 10.1093/plcell/koaf277

**Published:** 2025-11-21

**Authors:** Christian Damian Lorenzo

**Affiliations:** Assistant Features Editor, The Plant Cell, American Society of Plant Biologists; Center for Plant Systems Biology, VIB, Gent B-9052, Belgium; Department of Plant Biotechnology and Bioinformatics, Ghent University, Gent B-9052, Belgium

The green revolution (GR) represented a major breakthrough in modern agricultural practices. This transformative period was characterized by high-yielding crop varieties, enhanced fertilizer inputs, and novel irrigation systems, leading to an outstanding and unprecedented boost of crop production (Evans and Lawson 2020). Cereals were the main stars of the GR era, particularly wheat and rice semi-dwarf varieties, which exhibited decreased lodging and a good response to fertilizers. The short stature in bread wheat varieties was achieved by the introduction of 2 modified alleles, *reduced height-1*, *Rht1* (*Rht-B1b*), and *Rht2* (*Rht-D1b*), whose mechanisms of action were discovered years later (Peng et al. 1999). *Rht-B1* and *Rht-D1* are homoeologs, encoding a DELLA-type protein, a key regulator of plant growth controlled by the plant hormone gibberellin (GA). Generally, DELLAs act by modulating growth, for example, to allocate plant resources to withstand stress (Lantzouni et al. 2020). The biosynthesis of GA and binding to its receptor GIBBERELLIN INSENSITIVE DWARF1 (GID1) targets DELLAs for degradation, nullifying the restrictive effect and promoting growth and organ enlargement (Van De Velde et al. 2017). *Rht-B1b* and *Rht-D1d* mutant alleles encode DELLA variants with reduced affinity for the GA-GID1 complex, resulting in reduced-height plants. While the aboveground characteristics of semi-dwarf wheat have been extensively studied over the years, the belowground effects of these alleles remain poorly understood and have not yet been investigated at the population level.

Aiming to unearth more information about *Rht-B1b* and *D1d* roles in root development, Xiaoming Wang, Peng Zhao, Xue Shi, Xiaolong Guo, and collaborators (Wang et al. 2025) took on this task by performing a large-scale analysis of root phenotypes of several worldwide wheat varieties. The researchers developed a high-throughput pipeline (Fig.) for root analysis and systematically performed phenotyping assays on seedlings of 406 wheat varieties, measuring root length, diameter, and volume, among other parameters. They also performed bulk RNA-seq to analyze expression profiles and single-nucleotide polymorphisms (SNPs) variants of root tissue–expressed genes, thus assembling a complete genomic-transcriptomic-phenotypic dataset. Initial phylogenetic clustering of populations allowed the identification of 190 modern cultivars released after the GR and 87 landraces among all accessions. Further transcriptomic classification identified a set of differentially expressed genes between the 2 groups, suggesting that root transcriptomes were affected by GR-related selection.

**Figure.**
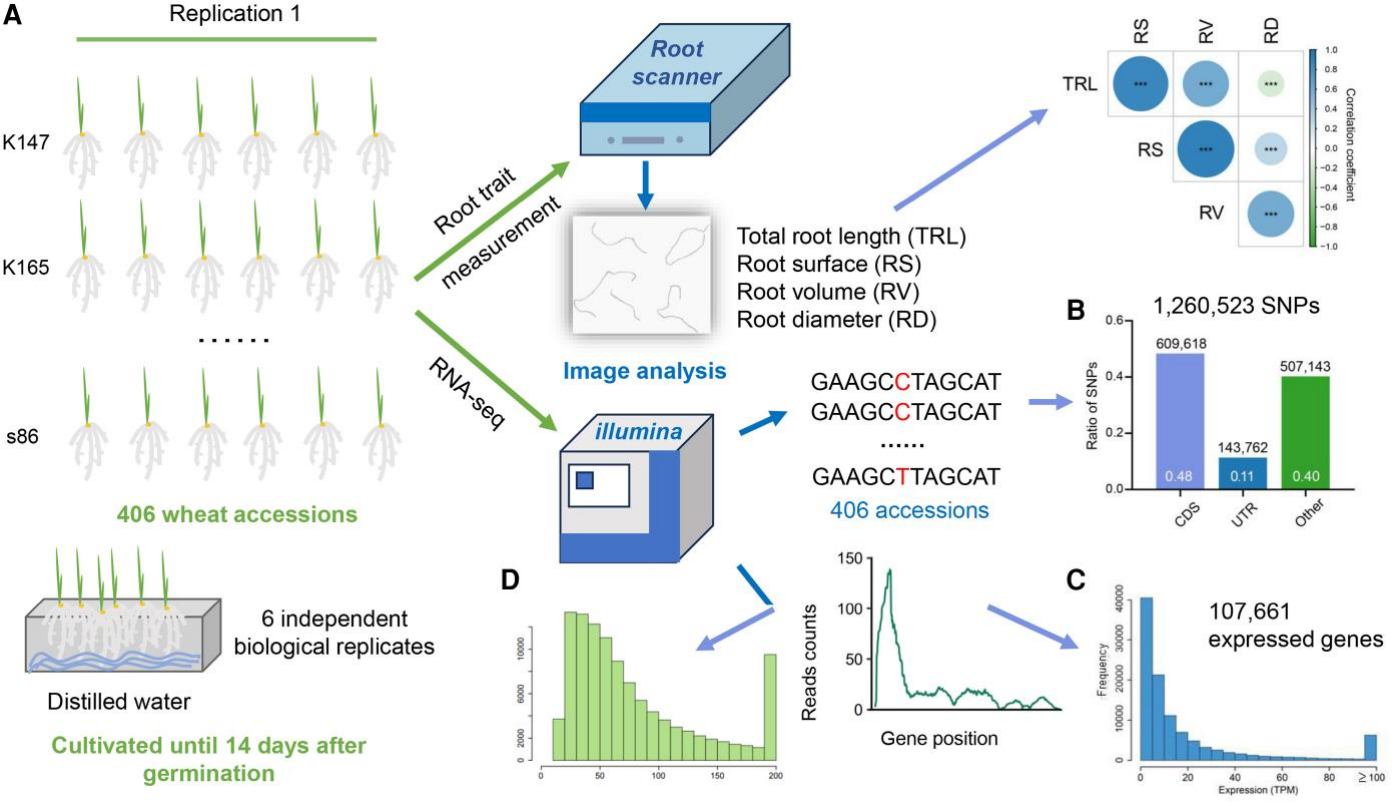
Schematic representation of the pipeline used to study root phenotypical changes at the genomic and transcriptomic level on wheat modern cultivars and landraces. **A)** Overview of the phenotyping and genotyping workflows. **B)** The distribution of the identified SNPs in gene regions. **C)** The transcripts per million (TPM) distribution of expressed genes. **D)** The coefficient of variation of expressed genes. Adapted from Wang et al. (2025) Figure 1.

Combining transcriptome-wide association study with genome-wide association study, researchers identified a common feature underlying part of the differences. The authors identified changes between *Rht-D1a* (the nonmutated allele of *Rht-D1*) and *Rht-D1b* (the GR allele) responsible for distinct root traits. Interestingly, the authors noticed that although the *Rht-B1b* mutant allele also contributes to dwarfism, it was not associated with root-related traits despite being a similar mutation to *Rht-D1b*. Experiments using near isogenic lines carrying *Rht-D1b* and *Rht-B1b* alleles, alongside assays with lines overexpressing each mutant allele under their native promoters, consistently confirmed the previous observation: while both mutant alleles reduce plant height, only *Rht-D1b* promotes root growth through increased meristematic length and width.

To further elucidate the molecular basis of *Rht-D1b*–mediated root trait regulation and its distinction from *Rht-B1b*, Wang and colleagues observed from RNA-seq data that lines carrying the *Rht-D1b* allele were less sensitive to the GA biosynthesis inhibitor paclobutrazol in root-related traits. Moreover, the cis-regulatory elements arrangements of both *Rht-B1* and *Rht-D1* were considerably different. Differential expression analyses also revealed largely non-overlapping sets of target genes, reinforcing the hypothesis of divergent downstream regulatory networks. Specifically, *Rht-D1d* affected pathways related to root meristem growth, cell cycle, and cell number regulation, while *Rht-B1b* was mostly associated with hormone homeostasis and reactive oxygen species regulation.

Overall, the study by Wang and colleagues uncovered a hidden legacy trait in modern wheat cultivars: a silent contributor to the high performance of semi-dwarf wheat varieties present since its incorporation during the GR. Future work might explore the molecular involvement of *Rht-D1b* in increased meristematic length and width.

## Recent related articles in *The Plant Cell*:

- Cheng and collaborators (Cheng et al. 2025) developed an integrated platform for multiplexed tissue sectioning and anatomical phenotyping and tested it in a diverse wheat population.
- Ke and collaborators (Ke et al. 2025) identified awn inhibitor gene *B1* as an important regulator influencing root hair length and nutrient uptake in wheat.

## Dive Curated Terms

The following phenotypic, genotypic, and functional terms are of significance to the work described in this paper:

GIBBERELLIN CHEBI: CHEBI:24250

initial AmiGo: PO:0004011
